# Gestational age at birth and morbidity, mortality, and growth in the first 4 years of life: findings from three birth cohorts in Southern Brazil

**DOI:** 10.1186/1471-2431-12-169

**Published:** 2012-10-31

**Authors:** Fernando C Barros, José Luis Diaz Rossello, Alicia Matijasevich, Samuel C Dumith, Aluisio J D Barros, Iná Silva dos Santos, Denise Mota, Cesar G Victora

**Affiliations:** 1 Postgraduate Course in Health and Behavior, Catholic University of Pelotas, Pelotas, Brazil; 2 Faculty of Medicine, Universidad de la Republica, Montevideo, Uruguay; 3 Postgraduate Program in Epidemiology, Federal University of Pelotas, Pelotas, Brazil; 4 Postgraduate Program in Health Science, Federal University of Rio Grande, Rio Grande, Brazil

**Keywords:** Gestational age, Preterm births, Early term births, Post-term births, Infant mortality, Neonatal mortality

## Abstract

**Background:**

We assessed anthropometric status, breastfeeding duration, morbidity, and mortality outcomes during the first four years of life according to gestational age, in three population-based birth cohorts in the city of Pelotas, Southern Brazil.

**Methods:**

Total breastfeeding duration, neonatal mortality, infant morbidity and mortality, and anthropometric measures taken at 12 and 48 months were evaluated in children of different gestational ages born in 1982, 1993 and 2004 in Southern Brazil.

**Results:**

Babies born <34 weeks of gestation and those born between 34–36 weeks presented increased morbidity and mortality, were breastfed for shorter periods, and were more likely to be undernourished at 12 months of life, in comparison with the 39–41 weeks group. Children born with 37 weeks were more than twice as likely to die in the first year of life, and were also at increased risk of hospitalization and underweight at 12 months of life. Post-term infants presented an increased risk of neonatal mortality.

**Conclusion:**

The increased risks of morbidity and mortality among preterm (<37 weeks of gestation) and post-term (>41 weeks) are well known. In our population babies born at 37 also present increased risk. As the proportion of preterm and early term babies has increased markedly in recent years, this is a cause for great concern.

## Background

During pregnancy, every week counts for growth and development, and the decision to interrupt gestation should be based on weighing the risks that the baby is facing in utero against the risks of preterm delivery.

The current definition of preterm births – less than 37 completed weeks of gestation, or three weeks before the date of delivery – was adopted by the World Health Organization [1] in 1975. The increased risks for preterm babies born before 34 weeks have long been established, and a number of studies have also shown late preterms (babies born between 34–36 weeks) present 3–5 times higher risk of dying than those born at term [2,3]. In this group, survivors are more likely to present cognitive deficiencies and neurologic impairments than children born at term [4-6]. At the other extreme of the scale, babies born after 41 weeks also present increased fetal and neonatal mortality, when compared with those born at term [7,8].

However, a number of recent studies from the United States and Europe show that babies born with 37 and 38 weeks of gestation also run increased risk, in comparison with those born with 39–41 weeks [9-15].

Because we were unable to find any studies from low or middle income countries on this topic, we decided to study the distribution by gestations age of several indicators of mortality, morbidity, anthropometric status and breastfeeding duration, with special emphasis on children born with 37 and 38 weeks of gestation. These outcomes were measured during the first four years of life in three population-based birth cohorts in the city of Pelotas in Southern Brazil.

## Methods

### Research setting and study design

During 1982, 1993 and 2004, birth cohorts representing all birth to mothers residing in the urban area of Pelotas, Southern Brazil, were enrolled. The three studies relied on primary data collection with the same methodology,[16-18] including recruitment of all hospital deliveries in the city (over 98% of all deliveries). Eligible mothers were interviewed soon after delivery using structured questionnaires covering socioeconomic variables, characteristics of pregnancy, labor, delivery and health care utilization. Similar variable definitions were used in all three studies. Non response rates at recruitment were below 1% in the three perinatal studies.

Data from the perinatal study and from follow-up home visits at 12 and 48 months were used in the present study. In the 1982 and 2004 cohorts, we attempted to locate all babies recruited in the perinatal phase. In the 1993 cohort we sought all low birth weight babies and a 20% sample of the remained; weighted data analyses were used to reproduce the total cohort [19].

Follow-up losses at 12 and 48 months visits were equal to 20.7% and 15.9% for the 1982 cohort, 6.6% and 12.8% for the 1993 cohort, and 5.9% and 8.2% for the 2004 cohort, respectively.

During each home visit mothers were interviewed by trained personnel who also conducted anthropometric measurements of the children. Tanita electronic scales (Tanita, Tokyo, Japan) with 100g precision were used for weight, the scales being calibrated on a weekly basis. Lengths and heights were measured with portable infantometers with 1mm precision. Birth length was not available for the 1982 birth cohort.

### Principal exposure (gestational age)

Gestational age estimation was based on the date of the last menstrual period (LMP). Cases of unknown or implausible LMP were treated as missing information. They corresponded to 15.6% of the births – 21% in 1982, 11% in 1933, and 7.3% in 2004. Gestational age was categorized as <34, 34–36, 37, 38, 39–41 and ≥42 completed weeks.

### Outcome data

Mortality surveillance was carried out actively through regular visits to all hospital, cemeteries, state vital registration services and the city's health department. Infant and neonatal mortality were defined as the deaths of live-born infants in the first 364 or28 days of live, respectively. Deaths in these periods were expressed per thousand live births: the infant (IMR) and neonatal mortality rates (NMR).

Information regarding breastfeeding was reported by the mother. Total breastfeeding duration (in months and days) was collected at each follow-up. The earliest available information on breastfeeding cessation was used to reduce recall bias.

Information on hospital admissions in the first 12 months of life was obtained from the mothers and was restricted to re-admissions after the newborn had been discharged from the maternity hospital.

The following anthropometric indices were calculated: weight-for-age (W/A), height-for-age (H/A) and weight-for-height (W/H) z-scores at 12 and 48 months, using the World Health Organization (WHO) growth standards [19]. Underweight was defined as W/A z-score below −2; stunting, as H/A z-score below −2; wasting, as W/H z-score below −2; and overweight, as W/A z-score above +2 standard deviations.

### Data on potential confounding factors

The following factors, measured in the perinatal period, were considered to be potential confounders of the association between gestational age and each outcome. Family income in the month prior to delivery was analyzed in quintiles; maternal schooling (completed years of formal education), maternal age (completed years) and height (in centimetres) were analyzed as continuous variables. Women who were single, widowed, divorced or lived without a partner were classified as single mothers. Parity was defined as the number of previous pregnancies resulting in a live birth or a late fetal death. Smoking during pregnancy, regardless of the number of cigarettes, was categorized as yes or no. Breastfeeding was not considered as a confounder, because it could not influence gestational age; in fact, it constitutes a potential mediator in the association between gestational age and the other outcomes.

### Statistical analyses

We tested heterogeneity between the three cohorts in terms of the association between gestational age and each outcome. Because no significant interactions were found (p<0.10), we pooled the data from the three cohorts.

We used *t*-test or *x*^2^ statistics to study crude associations between gestational age and each outcome. Confounder-adjusted analyses included logistic regression analysis for categorical outcomes and multiple linear regression for continuous outcomes. Analyses were carried out using Stata software, v. 11.0.

### Ethics

The study protocols of the three cohort studies were approved by the Medical Ethics Committee of the Federal University of Pelotas, which is affiliated with the Brazilian Federal Medical Council. In the 1982 and 1993 cohort studies, verbal consent to participate in the study was obtained from mothers. In the 2004 cohort study, mothers were provided written informed consent.

## Results

We examined separately the outcomes for each birth cohort, and the tables can be found as additional material files (Additional files 1, 2, 3). The results of the interactions between cohorts, in relation to each outcome are also available (Additional file 4).

Table 1 presents crude analyses of neonatal and infant mortality, hospitalizations in the first year of life, breastfeeding duration, and anthropometric measures at 12 and 48 months of age for different gestational age groups. Children born with less than 34 weeks of gestation presented the poorest outcomes in all respects, and those born between 39–41 weeks of gestation the best outcomes. Children born between 34–36 weeks presented increased neonatal and infant morbidity and mortality, increased rates of hospitalization, and greater weight and height deficits at 12 months of age. Children born at 37 weeks of gestation presented neonatal and infant mortality more than as high as the reference group of 39–41 weeks. Children born post-term children presented increased neonatal mortality, as compared with the 39–41 weeks reference group.

**Table 1:** Frequency of different outcomes during the first four years of life according to gestational age

**Outcome**	**Cohort**	**Number in the analyses**	**Gestational age in completed weeks**						**All**	**P value**
			**<34**	**34-36**	**37**	**38**	**39-41**	**42+**		
Neonatal mortality/1,000	All	13273	168	19	11	8	4	9	12	<0.001
Infant mortality/1,000	All	13273	189	40	23	14	10	17	21	<0.001
Total breastfeeding (months) (mean)	All	9453	6.1	7.5	8.4	8.3	8.1	7.7	8.0	0.004
Hospitalization 0–12 mo (%)	All	6071	38.1	26.1	18.0	16.6	14.3	15.2	17.1	<0.001
WAZ < −2 at 12 mo (%)	All	6059	6.8	4.5	2.7	2.0	1.5	2.8	2.3	<0.001
HAZ < −2 at 12 mo (%)	All	6048	14.5	10.3	6.6	6.1	5.6	6.3	6.6	<0.001
WHZ > 2 at 12 mo (%)	All	6048	4.7	6.4	10.4	8.5	8.4	7.8	8.2	0.06
WAZ < −2 at 48 mo (%)	All	8442	3.3	2.7	1.6	1.7	1.8	1.7	1.9	0.27
HAZ < −2 at 48 mo (%)	All	8442	7.9	6.8	5.4	5.4	6.5	7.5	6.4	0.29
WHZ > 2 at 48 mo (%)	All	8442	9.2	7.9	10.1	10.8	9.6	9.2	9.7	0.45
**Number of births in the 3 cohorts**	**All**		**869**	**1497**	**1434**	**2431**	**8287**	**1259**	**15777**	

Pelotas (Brazil) Birth Cohorts 1982, 1993, 2004.

Abbreviation: WAZ: weight for age z-score; HAZ: height for age z-score; WHZ: weight for height z-score.

Table 2 shows the adjusted analyses for these outcomes, controlled for family income, maternal education, age, and height, smoking during pregnancy, marital status, and parity. The poor outcomes of children born with less than 34 weeks were maintained for all outcomes. Compared to the 39–41 weeks reference groups, preterm babies <34 weeks and 34–36 weeks remained at increased risk of neonatal and infant mortality, were breastfed for a shorter period, were more hospitalized in the first year of life, and presented weight and height deficits at 12 months of life. Differences in weight and height were no longer observed at 48 months of age. Regarding children born with 37 weeks of pregnancy, neonatal and infant mortality rates were more than twice as high as those of babies with gestational ages between 39–41 weeks. This group was also more likely to be hospitalized in the first year of life, and to be underweight at 12 months of age. Post-term children presented a neonatal mortality more than twice as high as that for reference group. Finally, we did not find any disadvantages in children born with 38 weeks of gestation, as compared to those with 39–41 weeks.

**Table 2:** Adjusted* relative risks (for categorical variables) and beta coefficients (for numerical variables) of different outcomes according to gestational age (reference group= 39–41 weeks)

**Outcome**	**Gestational age in completed weeks**					
	**<34 (n=348)**	**34-36 (n=1202)**	**37 (n=1680)**	**38 (n=3089)**	**39-41 (n=7055)**	**42+ (n=745)**
Neonatal mortality (n=13031)	34.4 (21.6; 54.8)	3.4 (1.8; 6.6)	2.7 (1.3; 5.6)	2.0 (1.1; 3.5)	1.0	2.1 (1.0; 4.3)
Infant mortality (n=13031)	13.6 (9.7; 19.2)	3.1 (2.1; 4.7)	2.2 (1.4; 3.6)	1.4 (0.9; 2.2)	1.0	1.4 (0.9; 2.4)
Hospitalization 0–12 mo (n=5625)	2.4 (1.9; 3.1)	1.8 (1.4; 2.1)	1.2 (1.0; 1.6)	1.2 (1.0; 1.5)	1.0	0.9 (0.7; 1.2)
Breastfeeding (months) (n=5620)	−3.5 (−4.4; -2.4)	−0.8 (−1.5; -0.2)	−0.4 (−1.0; 0.3)	−0.4 (−0.9; 0.1)	0	−0.4 (−1.2; 0.3)
WAZ < −2 at 12 mo (%) (n=5615)	3.2 (1.7; 6.1)	2.6 (1.5; 4.5)	2.0 (1.1; 3.7)	1.4 (0.8; 2.5)	1.0	1.6 (0.8; 2.5)
HAZ < −2 at 12 mo (%) (n=5604)	2.0 (1.3; 2.9)	1.7 (1.2; 2.3)	1.3 (0.9; 1.9)	1.2 (0.9; 1.6)	1.0	1.0 (0.6; 1.5)
WHZ > 2 at 12 mo (%) (n=5604)	0.5 (0.2; 1.0)	0.7 (0.5; 1.0)	1.2 (0.9; 1.6)	1.0 (0.7; 1.2)	1.0	0.9 (0.6; 1.2)
WAZ < −2 at 48 mo (%) (n=7983)	1.4 (0.6; 3.1)	1.4 (0.8; 2.5)	0.9 (0.5; 1.7)	1.0 (0.6; 1.6)	1.0	0.8 (0.4; 1.5)
HAZ < −2 at 48 mo (%) (n=7946)	0.9 (0.5; 1.5)	1.0 (0.7; 1.4)	0.9 (0.6; 1.2)	0.8 (0.6; 1.1)	1.0	0.9 (0.6; 1.2)
WHZ > 2 at 48 mo (%) (n=7946)	1.1 (0.6; 1.8)	0.8 (0.6; 1.1)	1.0 (0.7; 1.2)	1.0 (0.8; 1.2)	1.0	1.0 (0.8; 1.3)

Pelotas (Brazil) Birth Cohorts 1982, 1993, 2004.

Abbreviation: WAZ: weight for age z-score; HAZ: height for age z-score; WHZ: weight for height z-score.

* Adjusted for family income, parity, smoking, marital status, height, maternal education, and maternal age.

## Discussion

Our study evaluated several outcomes in the first four years of age in three population-based birth cohorts in Southern Brazil. Although children in each cohort were born 11 years apart, we performed pooled analyses of the three cohorts because statistical tests did not show any evidence of interaction between each cohort and gestational age groups.

One limitation of this study, which is common in birth cohorts, was attrition rates in the follow-up visits. However, with the exception of the 12 months visit of the 1982, in which we failed to trace 20.7% of the children, we were able to locate at least 85% of the children in all other visits. Losses in 1982 were more frequent among the poorest and the richest strata of the population, as middle-class families were more easily found [17,20]. Also, as the estimation of gestational age was based on the date of the last menstrual period, we had 15.6% of missing cases.

We found that preterm babies, even those born between 34–36 weeks of gestation, children born with 37 weeks of gestation, and those born with 42 or more weeks were at increased risk of death in the first month of life, relative to children born between 39–41 weeks. Even after adjusting for possible confounders, compared to the reference groups of 39–41 weeks, the relative risks of neonatal death were more than 30 times higher for babies born before 34 weeks, 3.4 times higher for those born between 34–36 weeks, nearly three time higher for babies born at 37 weeks, and the double for post-term children. Preterm children and those born with 37 weeks of gestation were also at increased risk of infant mortality.

In addition to increased mortality risks, we found that all preterm children - including those born between 34–36 weeks - were more likely to present with stunting and underweight at 12 months of age, but this was no longer observed at 48 months of age. We also found that children born at 37 weeks were more likely to present underweight at 12 months of life. Post-term children presented increased risk of death in the first month of life.

Our finding of an increased risk of mortality and deficits in growth not only babies currently defined as preterm (<37 weeks), but also amongst those born at 37 weeks is a cause for concern. In Pelotas, where the whole gestational age curve has shifted to the left in the last three decades, and the prevalence of preterm births increased from 6.2% in 1982 to more than 15% in 2004 [21]. Births with 37 weeks also increased from 7.1% in 1982 to 11.4% in 2004. The causes for this increase in preterm births are not clearly elicited yet, but it is possible that interruption of pregnancies may have played an important role, as the proportion of cesarean sections doubled between 1982 and 2004 [21,22].

Finally, it is not possible to ascertain in an observational study if the increased risks observed among infants born before full maturity were due to early exposure to the extra-uterine environment, or damages produced by maternal conditions that may also have produced the untimely birth, or even a combination of both situations. A previous study suggests that preterm birth and exposure to maternal medical conditions are independent risk factors for neonatal morbidity, with the former playing a stronger role [11].

## Conclusions

This study supports the literature from high-income countries [9-15] in strongly suggesting that the current definition of preterm births – namely, deliveries occurring before 37 completed weeks of gestation - is no longer acceptable as an international standard to guide clinical practice. The need for a change in the cut-off has been already indicated [23,24]. After all, important researchers in the past, such as Lubchenco [25] and Tanner [26], proposed 38 weeks as the cut-off for preterm births.

## Abbreviations

H/A: Height-for-age z-score; IMR: Infant mortality rate; NMR: Neonatal mortality rate; W/A: Weight-for-age z-score; W/H: Weight-for-height z-scores; WHO: World Health Organization.

## Competing interests

The authors have indicated they have neither financial relationships relevant to this article to disclose nor any conflict of interests.

## Authors’ contributions

FCB, JLDR, and CGV contributed to the concept and the design of the study, and writing of the manuscript. AM, SCD, AJDB, ISS, and DM contributed to the analysis of the data. All authors contributed to the interpretation of the data and revising the manuscript, and final approval for publication. FCB and CGV are the guarantors. All authors read and approved the final manuscript.

## Pre-publication history

The pre-publication history for this paper can be accessed here:

http://www.biomedcentral.com/1471-2431/12/169/prepub

## Supplementary Material

Additional file 1 **Table 1. **Frequency of different outcomes during the first four years of life according to gestational age. Pelotas (Brazil) 1982 Birth Cohort. **Table 2. **Adjusted* relative risks (for categorical variables) and beta coefficients (for numerical variables) of different outcomes according to gestational age (reference group= 39-41 weeks, n=4496). Pelotas (Brazil) 1982 Birth Cohort. Click here for file

Additional file 2 **Table 1. **Frequency of different outcomes during the first four years of life according to gestational age. Pelotas (Brazil) 1993 Birth Cohort. **Table 2. **Adjusted* relative risks (for categorical variables) and beta coefficients (for numerical variables) of different outcomes according to gestational age (reference group= 39-41 weeks). Pelotas (Brazil) 1993 Birth Cohort. Click here for file

Additional file 3 **Table 1. **Frequency of different outcomes during the first four years of life according to gestational age. Pelotas (Brazil) 2004 Birth Cohort. **Table 2. **Adjusted* relative risks (for categorical variables) and beta coefficients (for numerical variables) of different outcomes according to gestational age (reference group= 39-41 weeks). Pelotas (Brazil) 2004 Birth Cohort. Click here for file

Additional file 4 **Table 1. **Number of cases analyzed for each outcome and p-value for the regression analyses for interactions between each outcome by cohort and gestational age groups. Pelotas (Brazil) Birth Cohorts 1982, 1993, 2004. Click here for file

## References

[B1] World Health Organization Manual of the international statistical classification of diseases, injuries and causes of death 1975 WHO, Geneva vol. 1 (9th Revision)

[B2] Kramer MS Demissie K Yang H Platt RW Sauve R Liston R The contribution of mild and moderate preterm birth to infant mortality Fetal and Infant Health Study Group of the Canadian Perinatal Surveillance System. Jama 2000 284 7 843 849 10.1001/jama.284.7.843 10938173

[B3] Santos IS Matijasevich A Silveira MF Sclowitz IK Barros AJ Victora CG Barros FC Associated factors and consequences of late preterm births: results from the 2004 Pelotas birth cohort Paediatr Perinat Epidemiol 2008 22 4 350 359 10.1111/j.1365-3016.2008.00934.x 18578748

[B4] Chyi LJ Lee HC Hintz SR Gould JB Sutcliffe TL School outcomes of late preterm infants: special needs and challenges for infants born at 32 to 36 weeks gestation J Pediatr 2008 153 1 25 31 10.1016/j.jpeds.2008.01.027 18571530

[B5] Petrini JR Dias T McCormick MC Massolo ML Green NS Escobar GJ Increased risk of adverse neurological development for late preterm infants J Pediatr 2009 154 2 169 176 10.1016/j.jpeds.2008.08.020 19081113

[B6] Moster D Lie RT Markestad T Long-term medical and social consequences of preterm birth N Engl J Med 2008 359 3 262 273 10.1056/NEJMoa0706475 18635431

[B7] De Los Santos-Garate AM Villa-Guillen M Villanueva-Garcia D Vallejos-Ruiz ML Murguia-Peniche MT Perinatal morbidity and mortality in late-term and post-term pregnancy. NEOSANO perinatal network's experience in Mexico J Perinatol 2011 31 12 789 793 10.1038/jp.2011.43 21681180

[B8] Altman M Edstedt Bonamy AK Wikstrom AK Cnattingius S Cause-specific infant mortality in a population-based Swedish study of term and post-term births: the contribution of gestational age and birth weight BMJ Open 2012 2 4 10.1136/bmjopen-2012-001152 PMC3391369 22763662

[B9] Cheng YW Nicholson JM Nakagawa S Bruckner TA Washington AE Caughey AB Perinatal outcomes in low-risk term pregnancies: do they differ by week of gestation? Am J Obstet Gynecol 2008 199 4 e371 377 370 10.1016/j.ajog.2008.08.008 18928977

[B10] McIntire DD Leveno KJ Neonatal mortality and morbidity rates in late preterm births compared with births at term Obstet Gynecol 2008 111 1 35 41 10.1097/01.AOG.0000297311.33046.73 18165390

[B11] Shapiro-Mendoza CK Tomashek KM Kotelchuck M Barfield W Nannini A Weiss J Declercq E Effect of late-preterm birth and maternal medical conditions on newborn morbidity risk Pediatrics 2008 121 2 e223 232 10.1542/peds.2006-3629 18245397

[B12] Zhang X Kramer MS Variations in mortality and morbidity by gestational age among infants born at term J Pediatr 2009 154 3 358 362 362 e351 10.1016/j.jpeds.2008.09.013 18950794

[B13] Lindstrom K Winbladh B Haglund B Hjern A Preterm infants as young adults: a Swedish national cohort study Pediatrics 2007 120 1 70 77 10.1542/peds.2006-3260 17606563

[B14] MacKay DF Smith GC Dobbie R Pell JP Gestational age at delivery and special educational need: retrospective cohort study of 407,503 schoolchildren PLoS Med 2011 7 6 e1000289 20543995 10.1371/journal.pmed.1000289 PMC2882432

[B15] Mathiasen R Hansen BM Andersen AM Forman JL Greisen G Gestational age and basic school achievements: a national follow-up study in Denmark Pediatrics 2010 126 6 e1553 1561 10.1542/peds.2009-0829 21059721

[B16] Santos IS Barros AJD Matijasevich A Domingues MR Barros FC Victora CG Cohort Profile: the 2004 Pelotas (Brazil) Birth Cohort Study Int J Epidemiol 2011 40 6 1461 1468 10.1093/ije/dyq130 20702597 PMC3235016

[B17] Victora CG Barros FC Cohort profile: the 1982 Pelotas (Brazil) birth cohort study Int J Epidemiol 2006 35 2 237 242 16373375 10.1093/ije/dyi290

[B18] Victora CG Hallal PC Araujo CL Menezes AM Wells JC Barros FC Cohort profile: the 1993 Pelotas (Brazil) birth cohort study Int J Epidemiol 2008 37 4 704 709 17846051 10.1093/ije/dym177

[B19] de Onis M Onyango AW Borghi E Siyam A Nishida C Siekmann J Development of a WHO growth reference for school-aged children and adolescents Bull World Health Organ 2007 85 9 660 667 10.2471/BLT.07.043497 18026621 PMC2636412

[B20] Victora CG Barros FC Lima RC Behague DP Gonçalves H Horta BL Gigante DP Vaughan JP The Pelotas birth cohort study, Rio Grande do Sul, Brazil, 1982–2001 Cad Saude Publ 2003 19 5 1241 1256 10.1590/S0102-311X2003000500003 PMC2841342 14666206

[B21] Barros FC Victora CG Barros AJ Santos IS Albernaz E Matijasevich A Domingues MR Sclowitz IK Hallal PC Silveira MF The challenge of reducing neonatal mortality in middle-income countries: findings from three Brazilian birth cohorts in 1982, 1993, and 2004 Lancet 2005 365 9462 847 854 10.1016/S0140-6736(05)71042-4 15752528

[B22] Cesar JA Matijasevich A Santos IS Barros AJ Dias-da-Costa JS Barros FC Victora CG The use of maternal and child health services in three population-based cohorts in Southern Brazil, 1982–2004 Cad Saude Publica 2008 24 Suppl 3 S427 436 18797718 10.1590/s0102-311x2008001500008

[B23] Clark SL Fleischman AR Term pregnancy: time for a redefinition Clin Perinatol 2011 38 3 557 564 10.1016/j.clp.2011.06.014 21890025

[B24] Kramer MS Papageorghiou A Culhane J Bhutta Z Goldenberg RL Gravett M Iams JD Conde-Agudelo A Waller S Barros F Challenges in defining and classifying the preterm birth syndrome Am J Obstet Gynecol 2011 206 2 108 112 22118964 10.1016/j.ajog.2011.10.864

[B25] Lubchenco L Searls D Brazie J Neonatal mortality rate: relationship to birth weight and gestational age J Pediatr 1972 81 4 814 822 10.1016/S0022-3476(72)80114-8 5074362

[B26] Tanner JM Foetus into Man 1978 Open Books, London 46

