# Supplementary material for: Gestational age at birth and morbidity, mortality, and growth in the first 4 years of life: findings from three birth cohorts in Southern Brazil
Source: BMC Pediatr. 2012 Oct 31;12:169. doi: 10.1186/1471-2431-12-169 (PMC3504558; doi:10.1186/1471-2431-12-169)
Supplement: Additional file 1 — Table 1. Frequency of different outcomes during the first four years of life according to gestational age. Pelotas (Brazil) 1982 Birth Cohort. Table 2. Adjusted* relative risks (for categorical variables) and beta coefficients (for numerical variables) of different outcomes according to gestational age (reference group= 39-41 weeks, n=4496). Pelotas (Brazil) 1982 Birth Cohort. [file 1471-2431-12-169-S1.docx]

**Table 1.** Frequency of different outcomes during the first four years of life according to gestational age. Pelotas (Brazil) 1982 Birth Cohort.

| Outcome | Number in the analyses | Gestational age in completed weeks | | | | | | **All** | P value |
| --- | --- | --- | --- | --- | --- | --- | --- | --- | --- |
|  |  | **<34** | **34-36** | **37** | **38** | **39-41** | **42+** |  |  |
| Neonatal mortality/1,000 | 5872 | 394 | 41 | 14 | 7 | 6 | 15 | 18 | <0.001 |
| Infant mortality/1,000 | 5872 | 477 | 90 | 32 | 17 | 16 | 25 | 33 | <0.001 |
| Total breastfeeding (months) (mean) | 5308 | 2.4 | 5.3 | 6.3 | 6.2 | 6.6 | 6.0 | 6.3 | 0.004 |
| Hospitalization 0-12 mo (%) | 1194 | 50.5 | 30.8 | 26.8 | 24.1 | 21.6 | 20.3 | 22.9 | 0.310 |
| WAZ < -2 at 12 mo (%) | 1190 | 0 | 10.8 | 4.9 | 2.5 | 1.7 | 3.4 | 2.7 | 0.001 |
| HAZ < -2 at 12 mo (%) | 1190 | 25.0 | 16.9 | 9.8 | 9.5 | 6.0 | 3.4 | 7.2 | 0.004 |
| WHZ > 2 at 12 mo (%) | 1190 | 0.0 | 3.1 | 7.3 | 7.5 | 7.4 | 3.4 | 6.7 | 0.480 |
| WAZ < -2 at 48 mo (%) | 3791 | 5.6 | 4.0 | 1.5 | 2.2 | 2.0 | 1.3 | 2.1 | 0.300 |
| HAZ < -2 at 48 mo (%) | 3791 | 22.2 | 11.9 | 9.4 | 9.4 | 9.4 | 9.0 | 9.6 | 0.460 |
| WHZ > 2 at 48 mo (%) | 3784 | 16.7 | 6.8 | 6.8 | 7.4 | 6.8 | 7.8 | 7.0 | 0.657 |
| **Number of births in the cohort** |  | **49** | **245** | **330** | **711** | **2868** | **471** | **4674** | **-** |

Abbreviation: WAZ: weight for age z-score; HAZ: height for age z-score; WHZ: weight for height z-score.

**Table 2.** Adjusted* relative risks (for categorical variables) and beta coefficients (for numerical variables) of different outcomes according to gestational age (reference group= 39-41 weeks, n=4496). Pelotas (Brazil) 1982 Birth Cohort.

| Outcome | Gestational age in completed weeks | | | | | |
| --- | --- | --- | --- | --- | --- | --- |
|  | **<34** | **34-36** | **37** | **38** | **39-41** | **42+** |
|  |  |  |  |  |  |  |
| Neonatal mortality (n=4596) | 75.1 (39.0; 144) | 3.4 (1.3; 8.7) | 2.7 (1.1; 7.0) | 1.3 (0.5; 3.4) | 1.0 | 2.1 (0.9; 5.1) |
| Infant mortality (4596) | 33.1 (19.9; 54.9) | 4.6 (2.7; 7.9) | 2.3 (1.2; 4.4) | 1.2 (0.6; 2.4) | 1.0 | 1.4 (0.7; 2.8) |
|  |  |  |  |  |  |  |
| Hospitalization 0-12 m (n=1180) | 3.7 (0.9; 15.4) | 1.5 (0.9; 2.4) | 1.1 (0.7; 1.8) | 1.2 (0.8; 1.7) | 1.0 | 0.8 (0.5; 1.3) |
|  |  |  |  |  |  |  |
| Breastfeeding (months) (n=1180) | -4.0 (-0.4; -2.5) | -1.1 (-0.1; -2.1) | -0.4 (-1.4; 0.5) | -0.3 (-1.0; 0.5) | 0 | -0.5 (-1.5; 0.5) |
|  |  |  |  |  |  |  |
| WAZ < -2 at 12 m (n=1176) | Not calculated | 6.7 (2.5; 17.7) | 3.3 (1.0; 10.4) | 1.9 (0.6; 5.5) | 1.0 | 1.5 (0.5; 49) |
| HAZ < -2 at 12 m (n=1176) | 3.5 (0.4; 26.3) | 2.6 (1.3; 5.2) | 1.6 (0.7; 3.6) | 1.6 (0.9; 2.8) | 1.0 | 0.5 (0.1; 1.4) |
| WHZ > 2 at 12 m (n=1176) | Not calculated | 0.4 (0.1; 1.7) | 1.0 (0.4; 2.3) | 0.9 (0.5; 1.6) | 1.0 | 0.5 (0.1; 1.4) |
|  |  |  |  |  |  |  |
| WAZ < -2 at 48 m (n=3731) | 1.9 (0.2; 14.4) | 1.2 (0.8; 1.9) | 0.9 (0.6; 1.4) | 0.9 (0.7; 1.3) | 1.0 | 0.8 (0.5; 1.1) |
| HAZ < -2 at 48 m (n=3731) | 1.6 (0.6-4.4) | 1.2 (1.2; 2.4) | 1.3 (1.0; 1.8) | 1.3 (1.0; 1.7) | 1.0 | 0.8 (0.5; 1.3) |
| WHZ > 2 at 48 m (n=3731) | 2.4 (0.7; 2.5) | 1.0 (0.5; 1.2) | 1.0 (0.6; 1.7) | 1.0 (0.7; 1.4) | 1.0 | 1.2 (0.8; 1.8) |
|  |  |  |  |  |  |  |

Abbreviation: WAZ: weight for age z-score; HAZ: height for age z-score; WHZ: weight for height z-score.

* Adjusted for family income, parity, smoking, marital status, height, education, and maternal age.
