# Supplementary material for: Gestational age at birth and morbidity, mortality, and growth in the first 4 years of life: findings from three birth cohorts in Southern Brazil
Source: BMC Pediatr. 2012 Oct 31;12:169. doi: 10.1186/1471-2431-12-169 (PMC3504558; doi:10.1186/1471-2431-12-169)
Supplement: Additional file 2 — Table 1. Frequency of different outcomes during the first four years of life according to gestational age. Pelotas (Brazil) 1993 Birth Cohort. Table 2. Adjusted* relative risks (for categorical variables) and beta coefficients (for numerical variables) of different outcomes according to gestational age (reference group= 39-41 weeks). Pelotas (Brazil) 1993 Birth Cohort. [file 1471-2431-12-169-S2.docx]

**Table 1.** Frequency of different outcomes during the first four years of life according to gestational age. Pelotas (Brazil) 1993 Birth Cohort.

| Outcome | Number in the analyses | Gestational age in completed weeks | | | | | | **All** | P value |
| --- | --- | --- | --- | --- | --- | --- | --- | --- | --- |
|  |  | **<34** | **34-36** | **37** | **38** | **39-41** | **42+** |  |  |
| Neonatal mortality/ 1,000 | 4672 | 126 | 13 | 13 | 11 | 3 | 6 | 9 | <0.001 |
| Infant mortality (%) | 4672 | 140 | 28 | 25 | 14 | 6 | 13 | 15 | <0.001 |
| Total breastfeeding (months) (mean) | 1238 | 5.5 | 6.6 | 8.5 | 7.3 | 8.3 | 9.3 | 8.1 | 0.180 |
| Hospitalization 0-12 mo (%) | 1243 | 16.7 | 9.3 | 9.2 | 6.1 | 7.0 | 3.9 | 7.1 | 0.115 |
| WAZ < -2 at 12 mo (%) | 1241 | 8.6 | 6.2 | 2.6 | 1.8 | 1.7 | 2.0 | 2.3 | 0.016 |
| HAZ < -2 at 12 mo (%) | 1241 | 16.7 | 16.5 | 6.5 | 7.9 | 7.4 | 8.5 | 8.5 | 0.028 |
| WHZ > 2 at 12 mo (%) | 1241 | 5.6 | 4.2 | 18.4 | 7.3 | 10.1 | 11.2 | 9.8 | 0.033 |
| WAZ < -2 at 48 mo (%) | 1122 | 2.9 | 4.4 | 1.4 | 1.4 | 2.2 | 0.0 | 2.0 | 0.282 |
| HAZ < -2 at 48 mo (%) | 1227 | 8.8 | 8.9 | 1.4 | 4.2 | 4.2 | 5.1 | 4.6 | 0.207 |
| WHZ > 2 at 48 mo (%) | 1217 | 5.9 | 5.6 | 13.9 | 9.2 | 11.9 | 10.3 | 10.8 | 0.379 |
| **Number of births in the cohort** |  | **143** | **393** | **315** | **621** | **2680** | **520** | **4672** | **-** |

Abbreviation: WAZ: weight for age z-score; HAZ: height for age z-score; WHZ: weight for height z-score.

**Table 2.** Adjusted* relative risks (for categorical variables) and beta coefficients (for numerical variables) of different outcomes according to gestational age (reference group= 39-41 weeks). Pelotas (Brazil) 1993 Birth Cohort.

| Outcome | Gestational age in completed weeks | | | | | |
| --- | --- | --- | --- | --- | --- | --- |
|  | **<34**  **(n=186)** | **34-36**  **(n=416)** | **37**  **(n=315)** | **38**  **(n=621)** | **39-41**  **(n=2680)** | **42+**  **(n=520)** |
| Neonatal mortality | 83.0 (37.0; 187) | 3.4 (1.1; 10.5) | 5.1 (1.7; 14.8) | 3.7 (1.3; 10.2) | 1.0 | 2.0 (0.5-7.7) |
| Infant mortality | 33.6 (18.7; 62.0) | 2.9 (1.4; 6.1) | 4.1 (2.0; 8.4) | 1.9 (0.9; 4.3) | 1.0 | 1.8 (0.7-4.3) |
|  |  |  |  |  |  |  |
| Hospitalization 0-12 mo | 1.7 (0.6; 4.3) | 1.0 (0.5; 2.0) | 1.0 (0.4; 2.3) | 1.0 (0.5; 1.9) | 1.0 | 0.4 (0.1-1.0) |
|  |  |  |  |  |  |  |
| Breastfeeding (months) | -2.6 (-0.5; -5.1) | -1.6 (-3.7; 0.5) | -0.5 (-3.8; 1.9) | 1.6 (-0.3; 0.2) | 0 | 0.6 (-1.6; 2.8) |
|  |  |  |  |  |  |  |
| WAZ < -2 at 12 mo | 4.1 (1.1; 15.2) | 2.8 (1.0; 7.6) | 2.3 (0.6; 8.3) | 1.3 (0.4; 4.5) | 1.0 | 1.3 (0.3-4.6) |
| HAZ < -2 at 12 mo | 1.5 (0.6; 4.1) | 2.3 (1.3; 4.0) | 1.2 (0.5; 2.7) | 1.1 (0.6; 2.0) | 1.0 | 1.0 (0.5-1.9) |
| WHZ > 2 at 12 mo | 0.5 (0.1; 2.1) | 0.4 (0.1; 1.1) | 2.2 (1.1; 4.2) | 0.7 (0.4; 1.4) | 1.0 | 1.1 (0.5-2.1) |
|  |  |  |  |  |  |  |
| WAZ < -2 at 48 mo | 0.3 (0.1; 5.2) | 1.7 (0.5; 5.3) | 1.7 (0.4; 6.4) | 1,1 (0.3; 3.7) | 1.0 | 0.1 (0.0-4.5) |
| HAZ < -2 at 48 mo | 1.5 (0.4; 5.5) | 1.8 (0.8; 3.9) | 1.3.(0.5; 3.7) | 0.8 (0.3; 2.2) | 1.0 | 1.2 (0.5-2.9) |
| WHZ > 2 at 48 mo | 0.5 (0.1; 2.2) | 0.3 (0.1; 0.9) | 1.1 (0.5; 2.5) | 0.7 (0.3; 1.4) | 1.0 | 0.9 (0.5-1.8) |
|  |  |  |  |  |  |  |

Abbreviation: WAZ: weight for age z-score; HAZ: height for age z-score; WHZ: weight for height z-score.

* Adjusted for parity, smoking, marital status, height, education, and maternal age.

.
