# Supplementary material for: Gestational age at birth and morbidity, mortality, and growth in the first 4 years of life: findings from three birth cohorts in Southern Brazil
Source: BMC Pediatr. 2012 Oct 31;12:169. doi: 10.1186/1471-2431-12-169 (PMC3504558; doi:10.1186/1471-2431-12-169)
Supplement: Additional file 3 — Table 1. Frequency of different outcomes during the first four years of life according to gestational age. Pelotas (Brazil) 2004 Birth Cohort. Table 2. Adjusted* relative risks (for categorical variables) and beta coefficients (for numerical variables) of different outcomes according to gestational age (reference group= 39-41 weeks). Pelotas (Brazil) 2004 Birth Cohort. [file 1471-2431-12-169-S3.docx]

**Table 1.** Frequency of different outcomes during the first four years of life according to gestational age. Pelotas (Brazil) 2004 Birth Cohort.

| Outcome | Number in the analyses | Gestational age in completed weeks | | | | | | **All** | P value |
| --- | --- | --- | --- | --- | --- | --- | --- | --- | --- |
|  |  | **<34** | **34-36** | **37** | **38** | **39-41** | **42+** |  |  |
| Neonatal mortality /1,000 | 4217 | 213 | 15 | 5 | 3 | 2 | 7 | 11 | <0.001 |
| Infant mortality /1,000 | 4217 | 214 | 21 | 12 | 10 | 7 | 11 | 19 | <0.001 |
| Total breastfeeding (months) (mean) | 3822 | 7.5 | 9.3 | 9.8 | 10.1 | 10.4 | 9.4 | 9.9 | <0.001 |
| Hospitalization 0-12 mo (%) | 3634 | 41.8 | 29.6 | 12.8 | 16.8 | 14.3 | 19.3 | 18.7 | <0.001 |
| WAZ < -2 at 12 mo (%) | 3628 | 6.7 | 3.1 | 2.3 | 1.9 | 1.3 | 3.0 | 2.1 | <0.001 |
| HAZ < -2 at 12 mo (%) | 3617 | 13.9 | 7.7 | 6.0 | 4.7 | 4.7 | 6.3 | 5.8 | <0.001 |
| WHZ > 2 at 12 mo (%) | 3622 | 4.6 | 7.5 | 9.6 | 9.1 | 8.1 | 7.8 | 8.2 | 0.362 |
| WAZ < -2 at 48 mo (%) | 3529 | 3.2 | 1.8 | 1.7 | 1.2 | 1.2 | 3.4 | 1.6 | 0.07 |
| HAZ < -2 at 48 mo (%) | 3535 | 6.3 | 4.1 | 3.6 | 2.0 | 3.1 | 6.3 | 3.5 | 0.006 |
| WHZ > 2 at 48 mo (%) | 3535 | 9.1 | 9.0 | 11.7 | 14.3 | 12.8 | 10.9 | 12.2 | 0.106 |
| **Number of births in the cohort** |  | **271** | **432** | **459** | **715** | **1796** | **303** | **3976** | **-** |

Abbreviation: WAZ: weight for age z-score; HAZ: height for age z-score; WHZ: weight for height z-score. .

**Table 2.** Adjusted* relative risks (for categorical variables) and beta coefficients (for numerical variables) of different outcomes according to gestational age (reference group= 39-41 weeks). Pelotas (Brazil) 2004 Birth Cohort.

| Outcome | Gestational age in completed weeks | | | | | |
| --- | --- | --- | --- | --- | --- | --- |
|  | **<34** | **34-36** | **37** | **38** | **39-41** | **42+** |
| Neonatal mortality | 83.9 (19.1; 367) | 6.9 (1.1; 41.3) | 2.0 (0.1; 22.8) | 2.5 (0.3; 17.9) | 1.0 | 3.2 (0.2; 35.8) |
| Infant mortality | 13.0 (6.2; 27.0) | 1.7 (0.6; 4.9) | 1.0 (0.3; 3.7) | 1.1 (0.3; 3.1) | 1.0 | 0.9 (0.2; 4.1) |
|  |  |  |  |  |  |  |
| Hospitalization 0-12 mo | 2.6 (2.0; 3.4) | 1.9 (1.5; 2.4) | 1.3 (1.0; 1.6) | 1.2 (1.0; 1.5) | 1.0 | 1.2 (0.9; 1.6) |
|  |  |  |  |  |  |  |
| Breastfeeding (months) | -5.1 (-6.7; -3.5) | -1.2 (-0.1; -2.3) | -0.7 (-1.6; 0.3) | -0.8 (-1.6; -0.1) | 0 | -0.8 (-0.2; 0.3) |
|  |  |  |  |  |  |  |
| WAZ < -2 at 12 mo | 3.8 (1.9; 7.7) | 2.1 (1.0; 4.3) | 1.8 (0.8; 3.9) | 1.5 (0.7; 3.1) | 1.0 | 1.7 (0.7; 3.9) |
| HAZ < -2 at 12 mo | 2.3 (1.5; 3.6) | 1.6 (1.0; 2.4) | 1.4 (0.9; 2.2) | 1.1 (0.7; 1.7) | 1.0 | 1.0 (0.6; 1.7) |
| WHZ > 2 at 12 mo | 0.5 (0.2; 1.0) | 0.8 (0.5; 1.3) | 1.2 (0.8; 1.7) | 1.1 (0.8; 1.5) | 1.0 | 0.9 (0.5; 1.5) |
|  |  |  |  |  |  |  |
| WAZ < -2 at 48 mo | 1.9 (0.7; 4.9) | 1.3 (0.5; 3.2) | 1.4 (0.6; 3.5) | 1.2 (0.5; 2.8) | 1.0 | 2.1 (0.9; 4.7) |
| HAZ < -2 at 48 mo | 1.5 (0.8; 2.9) | 1.2 (0.7; 2.2) | 1.4 (0.7; 2.5) | 0.7 (0.4; 1.4) | 1.0 | 1.4 (0.8; 2.5) |
| WHZ > 2 at 48 mo | 0.7 (0.4; 1.2) | 0.7 (0.5; 1.0) | 0.8 (0.6; 1.1) | 1.0 (0.8; 1.3) | 1.0 | 0.9 (0.6; 1.3) |
|  |  |  |  |  |  |  |
|  |  |  |  |  |  |  |

Abbreviation: WAZ: weight for age z-score; HAZ: height for age z-score; WHZ: weight for height z-score.

* Adjusted for parity, smoking, marital status, height, education, and maternal age.
