# Supplementary material for: Gestational age at birth and morbidity, mortality, and growth in the first 4 years of life: findings from three birth cohorts in Southern Brazil
Source: BMC Pediatr. 2012 Oct 31;12:169. doi: 10.1186/1471-2431-12-169 (PMC3504558; doi:10.1186/1471-2431-12-169)
Supplement: Additional file 4 — Table 1. Number of cases analyzed for each outcome and p-value for the regression analyses for interactions between each outcome by cohort and gestational age groups. Pelotas (Brazil) Birth Cohorts 1982, 1993, 2004. [file 1471-2431-12-169-S4.docx]

**Table 1.** Number of cases analyzed for each outcome and p-value for the regression analyses for interactions between each outcome by cohort and gestational age groups. Pelotas (Brazil) Birth Cohorts 1982, 1993, 2004.

| Outcome | Cohort | Number in the analyses | P value |
| --- | --- | --- | --- |
|  |  |  |  |
| Neonatal mortality (%) | All | 13273 | 0.229 |
| Infant mortality (%) | All | 13273 | 0.701 |
| Total breastfeeding (months) (mean) | All | 9335 | 0.687 |
| Hospitalization 0-12 mo (%) | All | 6071 | 0.560 |
| WAZ < -2 at 12 mo (%) | All | 6059 | 0.110 |
| HAZ < -2 at 12 mo (%) | All | 6048 | 0.153 |
| WHZ > 2 at 12 mo (%) | All | 6048 | 0.336 |
| WAZ < -2 at 48 mo (%) | All | 8442 | 0.410 |
| HAZ < -2 at 48 mo (%) | All | 8401 | 0.699 |
| WHZ > 2 at 48 mo (%) | All | 8401 | 0.240 |
| **Number of births in the 3 cohorts** | **All** | **15450** | **-** |

Abbreviation: WAZ: weight for age z-score; HAZ: height for age z-score; WHZ: weight for height z-score.
